# Association between Vitamin D and Dental Caries in a Sample of Canadian and American Preschool-Aged Children

**DOI:** 10.3390/nu13124465

**Published:** 2021-12-14

**Authors:** Tiffany L. Williams, Joseph Boyle, Betty-Anne Mittermuller, Caroline Carrico, Robert J. Schroth

**Affiliations:** 1 School of Dentistry, Virginia Commonwealth University, Richmond, VA 23284, USA; twilliams25@vcu.edu (T.L.W.); ckcarrico@vcu.edu (C.C.); 2 Department of Biostatistics, School of Medicine, Virginia Commonwealth University, Richmond, VA 23284, USA; boylejr@mymail.vcu.edu; 3 Department of Preventive Dental Science, Dr. Gerald Niznick College of Dentistry, University of Manitoba, Winnipeg, MB R3E 3P4, Canada; bmittermuller@chrim.ca; 4 Children’s Hospital Research Institute of Manitoba, Winnipeg, MB R3E 3P4, Canada; 5 Department of Pediatrics and Child Health, Max Rady College of Medicine, University of Manitoba, Winnipeg, MB R3E 3P4, Canada; 6 Section of Pediatric Dentistry, Winnipeg Regional Health Authority, Winnipeg, MB R3A 1R9, Canada; 7 Shared Health, Winnipeg, MB R3A 1R9, Canada

**Keywords:** early childhood caries, vitamin D, preschool children, dental caries, nutritional status, case–control studies, calcium, parathyroid hormone

## Abstract

Background: Inadequate vitamin D levels may increase the risk of caries during childhood. The purpose of this study was to investigate the association between 25-hydroxyvitamin D (25(OH)D) status and severe early childhood caries (S-ECC) in preschool children. Methods: Data were obtained from children <72 months of age in two case–control studies in Winnipeg, Manitoba and Richmond, Virginia. Serum analysis assessed 25(OH)D, calcium and parathyroid concentrations. Data on demographics, dental history and oral hygiene were obtained via questionnaires. Bivariate and multiple logistic regression analyses were performed to assess the relationships between demographic and biological variables and S-ECC. A *p*-value of ≤0.05 was significant. Results: Data were available for 200 children with S-ECC and 144 caries-free controls. Children with S-ECC had significantly lower 25(OH)D levels than those who were caries-free (*p* < 0.001), and children with deficient 25(OH)D levels were 10 times more likely to have S-ECC (*p* < 0.001). Multiple logistic regression revealed that having higher 25(OH)D and calcium concentrations (*p* = 0.019 and *p* < 0.0001, respectively), as well as being breastfed in infancy (*p* < 0.001), were significantly and independently associated with lower odds of S-ECC, while dental insurance (*p* = 0.006) was associated with higher odds of S-ECC. Conclusions: This study provides additional evidence of an association between nutritional status, specifically vitamin D and calcium levels, and S-ECC.

## 1. Introduction

There is increasing interest in the relationship between oral and systemic health. One such area that is garnering more awareness is the connection between caries and the nutritional status of children. Nutritional status can be assessed in different ways, including assessing body mass index (BMI), using validated nutritional questionnaires, using food frequency questionnaires and measuring serum concentrations of certain nutrients.

The relationship between nutrition and caries in children is not new. Vitamin D has long been considered an important factor that affects overall health and well-being including the oral health of children. In fact, some of the earliest research on the topic was first published nearly a century ago [1]. Pioneering work by Lady May Mellanby provided the first evidence that vitamin D deficiency was associated with dental caries in children [1]. More recently, there has been a growing body of evidence that low serum concentrations of 25-hydroxyvitamin D (25(OH)D) are associated with increased caries experience [2,3,4,5]. 

Early childhood caries (ECC) is one of the most commonly occurring diseases in young children worldwide, including North America [6]. It crosses all socioeconomic and ethnic groupings, but is particularly prevalent among children from inner-city communities, low-income households and access-to-care-deprived areas. Common risk factors for ECC exist, including improper infant feeding, exposure to sugars, inadequate oral hygiene, poverty and delayed access to preventive dental care [7,8]. Many prenatal and early childhood factors contribute to ECC risk and treatment of ECC [9]. Low 25(OH)D levels during pregnancy have even been reported to increase the risk of ECC in infancy [10]. 

Vitamin D might contribute to a child’s risk of caries in a few biologically plausible ways [3,11,12]. Serum 25(OH)D has also been implicated in the proper formation of the developing tooth bud [13]. Prenatal vitamin D deficiency during periods of primary tooth enamel formation may result in enamel hypoplasia, which is a known risk factor for ECC. Further, it may contribute to a lowered immune response because of decreased levels of cathelicidin and defensins: antimicrobial peptides that reduce the risk of developing dental caries by attacking cariogenic bacteria [11,12]. One other potential pathway is through altering the composition and flow of saliva, resulting in fewer calcium ions in saliva [11].

While low 25(OH)D levels may contribute to caries, vitamin D deficiency and other nutritional problems may also arise because severe decay and poor oral health status may affect a child’s ability to bite and chew [14]. This in turn would limit the types of foods they normally consume, resulting in softer, carbohydrate-filled diets in place of meats, fresh fruits and vegetables.

The purpose of this study was to investigate the association between vitamin D status and severe ECC (S-ECC) in preschool children from two regions of North America.

## 2. Materials and Methods

Ethics approval for this study was obtained from the Institutional Review Board for Human Subjects at Virginia Commonwealth University and the University of Manitoba’s Health Research Ethics Board. Study data were obtained from two similar case–control studies involving children <72 months of age, along with their parent or primary caregiver, that investigated the association between 25-hydroxyvitamin D (25(OH)D) and S-ECC. Children with S-ECC were considered cases, while controls were children who were caries-free. ECC and severe ECC were defined according to established clinical case definitions [15]. Combining datasets permitted us to explore whether there is an association between ECC and 25(OH)D levels in children <72 months of age, within two different regions of North America. For this specific study, the analysis sample was obtained by retrospectively combining these two datasets from two different academic centers. This was carried out for two reasons: to increase the generalizability of the results, as well as to create a larger sample with greater statistical power to detect effects due to smaller standard errors of test statistics and regression coefficients. Specifically, with a sample size of 143 children in each group, we had an 80% statistical power to detect a difference of 10 nmol/L in 25(OH)D levels between the S-ECC and caries-free groups, using a two-sample t-test, assuming a common standard deviation of 30 nmol/L and a 5% significance level. The first dataset was obtained from a sample of 90 children with available 25(OH)D serum levels from Richmond, Virginia (latitude 37.5° N) between November 2013 and December 2015 (60 children had S-ECC and 30 children were caries-free). The second dataset was from Winnipeg, Manitoba (latitude 49.9° N) comprising 261 children with available 25(OH)D results, recruited between November 2009 and July 2011 (140 children had S-ECC and 121 children were caries-free) [12,16]. Children with S-ECC were recruited on the day of their dental surgery, with venipunctures obtained while they were under general anesthesia. Caries-free children were recruited from primary care clinics, community dental clinics and the community in Richmond and Winnipeg. Serum analyses were conducted by the VCU Hospital Health Systems Clinical Pathology Laboratory team, using liquid chromatography and tandem mass spectroscopy. Analyses of 25(OH)D from samples obtained from children in Winnipeg were conducted by the Hospitals in Common Laboratory at Mount Sinai Hospital in Toronto, Canada, using Chemiluminescence Immunoassay, while the remaining analyses of calcium and parathyroid hormone (PTH) were performed by the Department of Biochemistry and Genetics Laboratory at Winnipeg’s Health Sciences Centre, using liquid chromatography. [12,16]

The two datasets contained numerous variables in common, and the combined sample retained variables common to both datasets. The combined dataset recorded information on the child’s nutritional status (e.g., 25(OH)D, calcium, PTH levels), health history, dental history, oral hygiene habits, demographics and parental characteristics. Children missing a 25(OH)D measurement were excluded from the combined sample. Units of certain biological measurements (e.g., 25(OH)D concentrations) that differed between datasets were converted as necessary (e.g., converting ng/mL to nmol/L), and levels of categorical variables were re-coded to retain as much information as possible. 

Thresholds for 25(OH)D levels were based on existing definitions; namely a level ≥ 75 nmol/L was considered optimal, concentrations >50 nmol/L was adequate/sufficient and <35 nmol/L was deficient [12,17,18]. Assays for 25(OH)D for children from Manitoba were conducted by Chemiluminescence Immunoassay, while liquid chromatography with mass spectrometry was used to analyze samples from children in Virginia. 

For the other covariates derived from questionnaires, having at least one parent with a college degree was considered a “higher” level of household education, a household income greater than USD 28,000 or CAD 28,000 per year was considered a “higher” level of income and a child’s estimate of brushing frequency being daily or better was considered “frequently”. For the definition of season, we considered summer as the period of May through October for both regions, based upon when 25(OH)D endogenous production is possible in these locations.

Descriptive statistics were calculated for all variables using means, standard deviations (SDs), counts and frequencies, as appropriate. Then, variables were compared between the S-ECC and caries-free cohorts. Continuous variables were compared between those with S-ECC and those who were caries-free using t-tests, while categorical variables were compared between cases and controls using chi-squared tests. Test statistics, degrees of freedom, odds ratios (ORs), 95% confidence intervals (CIs) and *p*-values were reported for these tests. 

Subsequently, child- and family-level covariates were tested for their association with S-ECC. Each covariate was tested separately in a univariate logistic regression model; those covariates with *p* < 0.1 in univariate analyses were included in a multiple logistic regression model for those with S-ECC. Certain demographic and nutritional status covariates that had substantive correlations with each other were omitted from consideration in multivariate analyses. Forwards and backwards selection was used to determine a final model, with improvement in Akaike’s Information Criterion (AIC) used as the criterion for continuing the algorithm. Variance inflation factors (VIFs) for variables in the final model were calculated in order to assess possible multicollinearity in the model. Variable coefficients, standard errors (SEs), test statistics and *p*-values were reported for models, along with their corresponding OR and 95% CI. All analyses were conducted in R version 3.6.1 (R Foundation, Vienna, Austria). Significance was set at *p* ≤ 0.05.

## 3. Results

Overall, data were available for 344 children: 144 were caries-free and 200 had S-ECC. As shown in Table 1, the overall mean age of participants was 42.1 ± 14.6 months, and there was a relatively equal distribution of children by sex, season of recruitment, household income and parental education. 

The mean 25(OH)D level of all children was 75.2 ± 30.7 nmol/L, which was in the optimal range. Table 1 presents the results on the association between caries status and 25(OH)D. Those with S-ECC had significantly lower mean 25(OH)D concentrations than those who were caries-free (69.6 ± 30.9 nmol/L vs. 82.9 ± 28.7, *p* < 0.001). When considering 25(OH)D threshold values, children with deficient 25(OH)D levels (i.e., <35 nmol/L) were significantly more likely to have S-ECC (OR = 10.1, *p* < 0.001) than children with optimal levels. Additionally, children with adequate 25(OH)D levels were significantly more likely to have S-ECC (OR = 1.8, *p* = 0.01) than children with optimal levels. Meanwhile, children with S-ECC had significantly higher PTH concentrations and lower calcium levels than caries-free children (Table 1). 

Several covariates were found to be significantly associated with S-ECC at the bivariate level (Table 1). Various demographic characteristics differed significantly between those with S-ECC and those who were caries-free, including mean age (*p* = 0.004), household income (*p* < 0.001), household education (*p* < 0.001), breastfeeding status (*p* < 0.001), season of draw (*p* = 0.042) and parental employment status (*p* < 0.001). 

Other significant associations with S-ECC were identified (Table 1). Children who were breastfed had lower odds of having S-ECC (OR = 0.33, *p* < 0.001), but no other prenatal, childhood health or infant feeding practice variable was significantly associated with S-ECC. Meanwhile, children who had already been to the dentist were 26.3 times more likely to have S-ECC (*p* < 0.001) and those with dental insurance were also significantly more likely to have S-ECC (*p* < 0.001).

All covariates which were significantly associated with S-ECC in the univariate analyses were combined into a multivariate model, omitting some which exhibited multicollinearity with other variables (e.g., income and education). Forward and backwards selection agreed on the same model, which is shown in Table 2. After controlling for season of draw, which is itself a marginally significant predictor of S-ECC status, increases in both 25(OH)D and calcium, as well as being breastfed as an infant (*p* < 0.001), are significantly and independently associated with a lower likelihood of S-ECC (*p* = 0.019 and *p* < 0.0001, respectively). Holding other covariates in the model constant, an increase in 25(OH)D was associated with a significant reduction in the odds of having S-ECC. For every 1 nmol/L increase in 25(OH)D, the odds of having S-ECC decreased by 1.04%. Having a method to pay for dental care was associated with a higher likelihood of S-ECC (*p* = 0.006). All VIF values for model coefficients were less than 1.1, which is much lower than any cutoffs, suggesting multicollinearity. 

## 4. Discussion

This study involved preschool-aged children recruited from two different North American centers, to investigate the relationship between S-ECC and 25(OH)D levels. A key finding from this study was that higher 25(OH)D concentrations were associated with reduced odds for caries, even after controlling for the influence of potential confounders. Since 25(OH)D levels can vary by season, we included the season in our regression modeling to control for any potential influence of endogenous 25(OH)D production. Overall, children in this study had mean 25(OH)D concentrations considered to be in the optimal range (i.e., ≥75 nmol/L). Despite these relatively good concentrations, we were still able to detect a difference between children with caries and those who were caries-free. Results from a national representative sample of Canadian school-aged children revealed that those with optimal concentrations had 39% lower odds for caries and overall caries experienced [19]. This appears to mirror conclusions from a notable review advising that 25(OH)D concentrations ≥ 75 nmol/L are protective against caries [20]. 

Our finding adds to the growing literature pointing to an association between low 25(OH)D and caries in children [4,10,12,21,22]. While most of these studies are cross-sectional in design, there is emerging evidence from prospective studies that also indicate an inverse relationship [10,22,23]. A recent vitamin D supplementation study involving a cohort of Canadian children from Winnipeg recently reported a significant inverse relationship between cord 25(OH)D levels and dt (i.e., number of decayed primary teeth) scores in infants [22]. An earlier birth cohort from this city also reported a significant association between low maternal prenatal 25(OH)D concentrations and ECC [10], and even found that prenatal calcium levels and enamel hypoplasia are significantly associated [24]. However, a recent Polish study investigating the relationship between parental reported vitamin D supplementation and caries did not identify a significant relationship [25]. 

A recent systematic review of 13 studies (many of high quality) concluded that there is evidence that suboptimal 25(OH)D concentrations (<75 nmol/L) are associated with an increased caries risk in children [5]. This review also suggested that low vitamin D levels should be considered as a potential risk factor for caries in children [5]. A recent Chinese study reported that children with optimal 25(OH)D levels had a significantly lower prevalence of ECC [21], while a small study from Iraq reported significantly higher 25(OH)D levels among caries-free preschool children [26]. However, another study revealed a statistically weak relationship between 25(OH)D status in early childhood and caries in primary teeth in children who were 6 years old [23]. A recent Spanish birth cohort study reported that low prenatal and childhood vitamin D levels were associated with caries in the permanent teeth of children [27].

The relationship between 25(OH)D and caries even extends beyond the primary dentition into the permanent dentition of children. A study of 7-year-olds in Portugal revealed that suboptimal 25(OH)D concentrations were significantly associated with advanced decay in permanent teeth [28]. A population-based prospective cohort study among mothers and children in the Netherlands reported that low maternal and neonatal 25(OH)D concentrations are associated with accelerated dental development of permanent teeth in children [29]. 

In addition to exploring the connection between caries and vitamin D, the results of this study also revealed that children with ECC had lower serum calcium concentrations than children who were caries-free. This mirrors observations from our study involving the Winnipeg sample of children, where children with S-ECC had significantly lower calcium concentrations than caries-free children, and where all cases of low calcium were exclusively found among children with S-ECC [12]. However, calcium was not included in the multiple regression modeling in that study over concerns of multicollinearity between 25(OH)D and calcium, as these variables are physiologically interrelated [12]. A recent Canadian prospective birth cohort study (with infant dental exams performed by an examiner blinded to maternal 25(OH)D levels) reported for the first time that maternal prenatal calcium levels are associated with enamel hypoplasia [24]. That study revealed that higher calcium concentrations were associated with lower odds for enamel hypoplasia [24]. These identified associations between vitamin D and calcium with caries and enamel hypoplasia are entirely plausible biologically. Vitamin D and calcium disturbances during tooth development may result in dentin and enamel defects, which can increase the risk of caries.

Our study identified that children with a history of breastfeeding had lower odds of having S-ECC. Other publications have provided evidence that breastfeeding in the first year of life is protective against caries or the treatment of S-ECC under general anesthesia [9,30,31,32]. A 2015 systematic review and meta-analysis concluded that breastfeeding is protective against caries during the early childhood period [33]. 

Interestingly, we found that the presence of dental insurance was associated with increased odds for S-ECC. This might be explained by the fact that S-ECC is heavily influenced by poverty and many of the children recruited with caries had government insurance intended for low-income populations. For instance, registered First Nations children in Canada have dental benefits, but because of numerous systemic and access-to-care challenges, they have some of the worst oral health conditions [34,35]. The same may be true for some of the children recruited at our US site. The presence of dental insurance does not guarantee early and regular access to preventive oral health care.

Over the last several years, the literature on the association between 25(OH)D and children’s oral health has grown [5,22,26,28,29,36]. One recent study reported that lower prenatal and neonatal concentrations of 25(OH)D were associated with an advanced dental age of children, suggesting that inadequate 25(OH)D accelerates dental development [29]. Lower prenatal 25(OH)D and calcium levels have been associated with ECC and enamel hypoplasia, respectively, in infants [10,24]. Others suggest an association between lower 25(OH)D concentrations among children with caries [12,26], which is confirmed by a recent systematic review [5]. While prenatal supplementation may reduce the risk for ECC, it is important that it is delivered in an appropriate amount that will benefit developing teeth, as not all supplementation has resulted in a reduced incidence of ECC [22,36]. It is clear that a relationship exists, underscoring the importance that future studies should measure prenatal exposure to vitamin D and nutrition and prospectively follow the child to assess the development of dental caries.

This study is not without limitations. Data from this case–control study were cross-sectional in nature, meaning that we were only able to investigate an association between 25(OH)D and caries and not causation. It is difficult to determine the exact nature of the relationship of vitamin D with early childhood caries given the multitude of variables that may contribute to both conditions: vitamin D deficiency and tooth decay. Some of the variables were obtained by questionnaires completed by parents, which are subject to recall and response bias. Unfortunately, we had limited information in common between the two datasets relating to ethnicity and the dietary intake of the children. This precluded us from assessing connections between intake of vitamin-D-rich or vitamin-D-fortified foods and 25(OH)D and caries status and reporting on ethnicity. Our study also involved participants from two different regions at different latitudes, which can influence the endogenous production of vitamin D. Fortunately, we controlled for season of collection in our final analyses. Regardless of such limitations, this study has strengths, including being novel and involving a relatively robust sample size of children from two regions of North America.

## 5. Conclusions

This study revealed a significant and independent association between caries and 25(OH)D status. Specifically, those with S-ECC had significantly lower concentrations of 25(OH)D than caries-free children, and those with optimal 25(OH)D levels had lower odds for S-ECC. Other significant associations identified with S-ECC included lower calcium levels and not being breastfed in infancy. This study provides additional evidence of an association between nutritional status, specifically vitamin D and calcium levels, and S-ECC.

## Figures and Tables

**Table 1:** Association between caries status and child and caregiver characteristics and nutritional measures.

Variable		Total N (%)	S-ECC N (%)	Caries-free N (%)	Odds Ratio (95% CI)	*p*-Value
Demographics:						
Location:	Winnipeg, MB	254 (74%)	140 (55%)	114 (45%)	0.61 (0.37, 1.02)	0.074
	Richmond, VA	90 (26%)	60 (67%)	30 (33%)	REF	
Age in months:		42.14 (14.55)	44.11 (12.70)	33.39 (16.44)	-	0.004 **
Season:	Winter	176 (51%)	93 (46%)	83 (58%)	0.64 (0.41, 0.98)	0.042
	Summer	168 (49%)	107 (54%)	61 (42%)	REF	
Sex:	Male	132 (52%)	70 (50%)	62 (54%)	0.84 (0.51, 1.38)	0.57
	Female	122 (48%)	70 (50%)	52 (46%)	REF	
Income:	>USD/CAD 28,000/year	165 (52%)	63 (35%)	102 (73%)	0.20 (0.12, 0.32)	<0.001 ***
	<USD/CAD 28,000/year	155 (48%)	117 (65%)	38 (27%)	REF	
Education:	One parent college of greater	170 (49%)	65 (33%)	105 (73%)	0.18 (0.11, 0.29)	<0.001 ***
	Neither college nor greater	174 (51%)	135 (67%)	39 (27%)	REF	
Employment:	At least one parent employed	237 (71%)	120 (60%)	117 (81%)	0.35 (0.21, 0.57)	<0.001 ***
	Neither parent employed	97 (20%)	80 (40%)	27 (19%)	REF	
Nutritional measures:						
Calcium (mg/dL):		9.46 (0.39)	9.33 (0.38)	9.63 (0.34)	-	<0.001 ***
PTH (pg/mL):		51.76 (27.56)	60.75 (27.00)	39.26 (23.15)	-	<0.001 ***
25(OH)D (nmol/L):		75.18 (30.69)	69.63 (30.94)	82.88 (28.71)	-	<0.001 ***
25(OH)D status:	Deficient (<35 nmol/L)	21 (6)	19 (10%)	2 (1%)	10.11 (2.28, 44.88)	<0.001 ***
	Inadequate (35–50 nmol/L)	32 (9%)	21 (10%)	11 (8%)	2.03 (0.92, 4.49)	0.08
	Adequate (50–75 nmol/L)	132 (38%)	83 (41%)	49 (34%)	1.80 (1.13, 2.89)	0.01 *
	Optimal (≥75 nmol/L)	159 (46%)	77 (39%)	82 (57%)	REF	
Prenatal history, childhood health and infant feeding practices:						
Premature:	Yes	45 (13%)	26 (13%)	19 (13%)	0.98 (0.52, 1.58)	0.96
	No	299 (87%)	174 (87%)	125 (87%)	REF	
Pregnancy complications:	Yes	62 (18%)	33 (17%)	29 (20%)	0.78 (0.45, 1.36)	0.39
	No	282 (82%)	167 (83%)	115 (80%)	REF	
Vitamin D supplementation:	Yes	26 (8%)	14 (7%)	12 (8%)	0.83 (0.37, 1.85)	0.65
	No	318 (92%)	186 (93%)	132 (92%)	REF	
Breastfed:	Yes	213 (62%)	103 (52%)	110 (76%)	0.33 (0.20, 0.53)	<0.001 ***
	No	131 (38%)	97 (48%)	34 (24%)	REF	
Bottle-fed:	Yes	224 (65%)	134 (67%)	90 (63%)	1.22 (0.78, 1.91)	0.39
	No	120 (35%)	66 (33%)	54 (37%)	REF	
Asthma:	Yes	49 (14%)	32 (16%)	17 (12%)	1.42 (0.76, 2.68)	0.27
	No	295 (86%)	168 (84%)	127 (88%)	REF	
Dental history and oral hygiene behaviors:						
Floss:	Yes	121 (35%)	70 (35%)	51 (35%)	0.98 (0.63, 1.54)	0.94
	No	223 (65%)	130 (65%)	93 (65%)	REF	
Brush at least daily:	Yes	294 (85%)	165 (83%)	129 (90%)	0.55 (0.29, 1.05)	0.07
	No	50 (15%)	35 (17%)	15 (10%)	REF	
Been to dentist before:	Yes	281 (82%)	195 (98%)	86 (60%)	26.3 (10.2, 67.9)	<0.001 ***
	No	63 (18%)	5 (2%)	58 (40%)	REF	
Method of paying for dental care:	Yes	307 (89%)	188 (94%)	119 (83%)	3.30 (1.59, 6.80)	<0.001 ***
	No/not sure	37 (11%)	12 (6%)	25 (17%)	REF	
Other:						
Sun exposure:	Yes	314 (91%)	181 (91%)	133 (92%)	0.79 (0.36, 1.71)	0.55
	No/not sure	30 (9%)	19 (9%)	11 (8%)	REF	

* *p* < 0.05; ** *p* < 0.01; *** *p* < 0.001. REF = reference category, USD = US dollar, CAD = Canadian Dollar, - = odds ratios not calculated for continuous data.

**Table 2:** Summary of logistic regression model for S-ECC.

Variable	Estimate	SE	Z	*p*-Value	Odds Ratio	95% CI
(Intercept)	23.198	3.860	6.01	<0.0001 ***	-	-
25(OH)D	−0.011	0.004	−2.33	0.019 *	0.989	(0.980, 0.998)
Calcium	−2.333	0.404	−5.77	<0.001 ***	0.097	(0.042, 0.208)
Breastfed	−1.067	0.283	−3.77	<0.001 ***	0.344	(0.195, 0.595)
Winter season	−0.833	0.273	−3.06	0.002 **	0.435	(0.252, 0.736)
Dental insurance method	1.224	0.445	2.75	0.006 **	3.402	(1.463, 8.517)

* *p* < 0.05; ** *p* < 0.01; *** *p* < 0.001. SE = standard error, Z = Z value, CI = confidence intervals, - = odds ratio and 95% CI not reported for intercept.

## Data Availability

Data are available on request due to restrictions. The data are not publicly available due to a data-sharing agreement between the University of Manitoba and the Virginal Commonwealth University.
